# Cytotoxic Potential of the Coelomic Fluid Extracted from the Sea Cucumber *Holothuria tubulosa* against Triple-Negative MDA-MB231 Breast Cancer Cells

**DOI:** 10.3390/biology8040076

**Published:** 2019-10-09

**Authors:** Claudio Luparello, Debora Ragona, Dalia Maria Lucia Asaro, Valentina Lazzara, Federica Affranchi, Monica Celi, Vincenzo Arizza, Mirella Vazzana

**Affiliations:** Dipartimento di Scienze e Tecnologie Biologiche, Chimiche e Farmaceutiche (STEBICEF), Università di Palermo, Viale delle Scienze, 90128 Palermo, Italy; debora0410ale@gmail.com (D.R.); daliaasaro@libero.it (D.M.L.A.); valentina.lazzara@community.unipa.it (V.L.); federica.affranchi@community.unipa.it (F.A.); monica.celi@unipa.it (M.C.); vincenzo.arizza@unipa.it (V.A.); mirella.vazzana@unipa.it (M.V.)

**Keywords:** *Holothuria tubulosa*, coelomic fluid, breast cancer, cell viability, cell cycle, mitochondrial function, autophagy

## Abstract

Growing evidence has demonstrated that the extracts of different holothurian species exert beneficial effects on human health. Triple negative breast cancers (TNBC) are highly malignant tumors that present a poor prognosis due to the lack of effective targeted therapies. In the attempt to identify novel compounds that might counteract TNBC cell growth, we studied the effect of the exposure of the TNBC cell line MDA-MB231 to total and filtered aqueous extracts of the coelomic fluid obtained from the sea cucumber *Holoturia tubulosa*, a widespread species in the Mediterranean Sea. In particular, we examined cell viability and proliferative behaviour, cell cycle distribution, apoptosis, autophagy, and mitochondrial metabolic/cell redox state. The results obtained indicate that both total and fractionated extracts are potent inhibitors of TNBC cell viability and growth, acting through both an impairment of cell cycle progression and mitochondrial transmembrane potential and a stimulation of cellular autophagy, as demonstrated by the increase of the acidic vesicular organelles and of the intracellular protein markers beclin-1, and total LC3 and LC3-II upon early exposure to the preparations. Identification of the water-soluble bioactive component(s) present in the extract merit further investigation aiming to develop novel prevention and/or treatment agents efficacious against highly metastatic breast carcinomas.

## 1. Introduction

Holothurians, also named sea cucumbers, belong to the class Holothuroidea (phylum Echinodermata), which includes around 1250 described living species worldwide. Holothurians have been widely studied in the last two decades for their high nutritious value and beneficial effects on human health [1,2,3], which have made them an object of investigation for possible therapeutic uses. In fact, the efficacy of holothurians to alleviate a number of different pathologies (asthma, rheumatism, hypertension, impotence, and others) is well known from Asian traditional medicine, as reported in the literature [4,5]. Furthermore, growing evidence has demonstrated that the extracts of different holothurian species are endowed with several varied biological and pharmacological properties, spanning from anti-angiogenic and anticancer to antimicrobial and anticoagulant activities (e.g., [1,6,7]). Such effects have been ascribed to the presence of numerous bioactive compounds among which the most characterized to date are the triterpene glycosides saponins, accounting for more than 300 different molecules, along with components of various macromolecular nature, such as glycosaminoglycans, phenolics, and peptides [8]. 

Triple-negative breast cancers (TNBC) are “aggressive” and highly malignant tumors characterized by the lack of expression of estrogen-, progesterone-, and epidermal growth factor type 2 (HER2) receptors, thereby being poorly responsive to hormonal therapies and to HER2-targeting drugs. This makes the TNBC histotype usually associated with worse prognosis than other breast tumors [9]. The limitation of treatment options against TNBC has prompted multiple approaches attempting to identify novel compounds that might counteract neoplastic cell growth. On the other hand, a thorough biological characterization of such compounds and the elucidation of their molecular mechanism of action is a necessary aspect for the development of targeted therapies. 

The sea cucumber *Holothuria tubulosa* is a widespread species in the Mediterranean Sea and one of the main species receiving rising commercial interest, being a seafood rich with nutrients and suitable for human consumption [10]. Dealing with biomedical applications, methanol extracts of this marine invertebrate were shown to restrain the inflammatory process in experimentally oedematous mice [11]. More recent studies have highlighted that coelomocytes, the immune mediator cells contained in the coelomic fluid (CF) of the animal, represent a source of antimicrobial substances and diffusible haemolytic factors active on rabbit and sheep erythrocytes [12,13]. These findings prompted an expansion of the investigation on the pharmacological potential of *H. tubulosa*-derived components, whose massive procurement represents an easy task due to the extensive dissemination of this aquatic organism.

In the present study, we examined the effect of the exposure of the TNBC cell line MDA-MB231 to total and filtered aqueous extracts of the CF obtained from *H. tubulosa*. The cell line chosen as the model system for this investigation is derived from a pleural effusion of a TNBC of basal subtype and displays several characteristics related to a highly malignant phenotype, such as the inactivation of p53 due to a mutation in codon 280 of exon 8, and the ability to form metastases in vivo [14,15,16]. In particular, our goal was to examine in a comprehensive fashion the CF extract-mediated modulation, if present, of viability and proliferative behaviour, cell cycle distribution, apoptosis and autophagy events, and mitochondrial metabolic/cell redox state. The results obtained indicate that both total and fractionated CF extracts are potent inhibitors of TNBC cell growth acting through both an impairment of cell cycle progression and mitochondrial respiration and a stimulation of cellular autophagy.

## 2. Materials and Methods

### 2.1. Catching and Safekeeping of the Animals 

**Figure 1:**
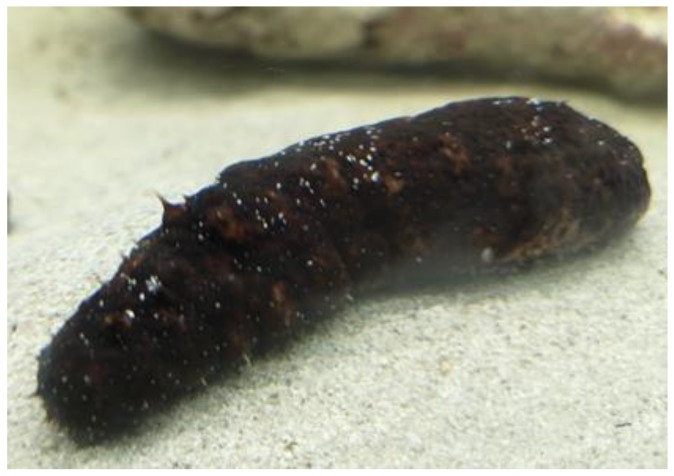
A specimen of *Holoturia tubulosa* sea cucumber.

A sample of 120 mature sea cucumbers (*Holothuria tubulosa*, Figure 1), approximately 11 ± 0.98 cm in length with a body weight of 46 ± 7.5 g, was collected from the Gulf of Palermo (Sicily, Italy) at a depth of 5–10 m, near a grassland of *Posidonia oceanica*, and the animals were manipulated after 2 weeks of acclimation in an aquarium filled with artificial sea water (0.425 M NaCl; 9 mM KCl; 9.3 mM CaCl_2_·2H_2_O; 0.0255 M MgSO_4_·7H_2_O; 0.023 MgCl_2_·6H_2_O; 2 mM NaHCO_3_ pH 8.0) at 15 ± 2 °C with constant oxygenation. The animals were fed with commercial invertebrate food (Algamac 3000, Aquafauna Bio-Marine Inc., Hawthorne, CA, USA). 

### 2.2. Bleeding Procedure and Preparation of CF Extracts

The animals were cut making an incision measuring 3–5 cm on the anterior-dorsal side using a scalpel, paying specific attention not to injure the internal organs. The CF was collected in several plastic beakers covered by a filter, kept on ice, and then transferred to polycarbonate tubes and immediately centrifuged at 1000 g for 10 min at 4 °C to remove the coelomocytes. Once separated from the cellular component, an aliquot of CF was stored at −80 °C and the remaining volume was filtered using Corning Spin-X UF 6 concentrators equipped with polyethersulfone membranes (10K MWCO; Corning Inc., Tewksbury, MA, USA). The tubes were centrifuged at maximum speed (4000 g) for approximately 3–4 h at 4 °C, and the filtered CF was stored at −80 °C. Then, both the total and filtered CFs, hereafter referred to as total extract and 10K fraction, respectively, were lyophilized in an Alpha 2–4 LD plus freeze-dryer (Martin Christ, Osterode am Harz, D). Aliquots of total extracts and 10K fractions were resuspended in the minimum volume of sterile distilled water and the protein concentration was measured with the Qubit Protein Assay Kit in the Qubit 3.0 fluorometer (ThermoFisher, Waltham, MA, USA), according to the manufacturer’s instructions. 

### 2.3. Cell Cultures

MDA-MB231 TNBC cells were cultured in D-MEM medium purchased from Sigma, St. Louis, MO, USA, supplemented with 10% foetal calf serum (FCS; ThermoFisher) and antibiotic/antimycotic mixture (100 U/mL penicillin, 100 μg/mL streptomycin, and 2.5 mg/L amphotericin B; ThermoFisher) at 37 °C in a 5% CO_2_ atmosphere. 

### 2.4. MTT Assay

Cell viability was assessed by an MTT assay [17]. Briefly, MDA-MB231 cells in exponential growth were plated at a concentration of 5500 cells/well in a 96-well plate, allowed to adhere overnight, and then treated with different concentrations of total extracts and 10K fractions for either 24 or 48 h. After addition of MTT and incubation with the solubilization buffer, the absorbance of the dissolved formazan was measured in an automated microplate reader (λ = 550 nm). Cell viability ratio between treated cells and controls was determined and the half maximal inhibitory concentrations (IC_50_) evaluated with the online IC_50_ calculator available at https://www.aatbio.com/tools/ic50-calculator (accessed June 2019).

### 2.5. Flow Cytometry

Flow cytometric assays were performed on treated and control cells as described in [18,19], using a FACSCanto instrument (BD Biosciences, Franklin Lakes, NJ, USA). All the data were analyzed with Flowing Software v.2.5.1. Gating in the FSC vs. SSC plot was performed before the specific analyses to exclude debris, which displayed low FSC values.

For the analysis of cell cycle distribution, cells were fixed with cold 70% ethanol, incubated with 40 μg RNase A/mL, and stained with 20 μg propidium iodide/mL. 

The externalization of phosphatidylserine, a hallmark of apoptotic events, was checked with the Annexin V-FITC kit (Canvax Biotech, Cordoba, Spain) according to the manufacturer’s instructions. 

The transmembrane mitochondrial potential (MMP) was checked using the mitochondria-selective dye JC1 (Molecular Probes, Eugene, OR, USA), which undergoes a fluorescence emission shift from green (~529 nm) to red (~590 nm) in case of physiologic MMP, whereas, in case of loss of MMP, a decrease in the red/green fluorescence intensity ratio can be observed. A valinomycin-treated positive control was included in the analysis.

The production of reactive oxygen species (ROS) was evaluated using the ROS Detection Assay Kit (Canvax Biotech) following the manufacturer’s instructions. A H_2_O_2_-treated positive control was included in the analysis.

The modulation of the autophagic behavior was checked via the flow cytometric quantification of i) the acidic vesicular organelles (AVOs), and ii) the autophagic markers beclin-1 and microtubule-associated protein light chain-3 (LC3), both total and as LC3-II form. For i), after cell fixing with cold 70% ethanol, cells were stained with 100 μg acridine orange/mL (Sigma) for 20 min in the dark. For ii), two different procedures were followed in the case of either beclin-1 and total LC-3 or LC3-II quantitation. For beclin-1 and total LC3 detection, cells were fixed with HistoChoice (Amresco, Solon, OH, USA) for 30 min at ambient temperature, washed with 2% FCS-containing phosphate-buffered saline (PBS), and incubated with the primary antibody dissolved in 0.1% Triton X-100 + 2% FCS + 40 μg propidium iodide/ml in PBS for 30 min. The antibodies used in the present study were rabbit polyclonal anti-beclin-1 (H-300, sc-11427, Santa Cruz Biotechnology, Dallas, TX, USA; working dilution 1:50, as recommended by He et al. [20]) and rabbit polyclonal anti-LC3 (L8918, Sigma; working dilution 1:250). Negative controls were prepared with the omission of the primary antibody. After exhaustive washing, the samples were incubated for 30 min with the secondary antibody (FITC-conjugated goat anti-rabbit IgG whole molecule, F0382, Sigma; working dilution 1:80), washed three times in PBS, and submitted to flow cytometry. For LC3-II detection, according to the procedure described by Eng et al. [21], unfixed cells were rinsed with PBS + 0.05% saponin (Sigma) to allow the extraction of the non-autophagosome-associated soluble LC3-I, and then incubated with the primary and secondary antibodies as previously reported in this paragraph, with the exception that saponin was always present in the antibody dilution buffers and replaced Triton X-100. 

### 2.6. Statistics

Statistics were checked through ANOVA test with SigmaPlot 11.0 software (SYSTAT, San Jose, CA, USA). A *p*-value < 0.05 was considered statistically significant.

## 3. Results

**Figure 2:**
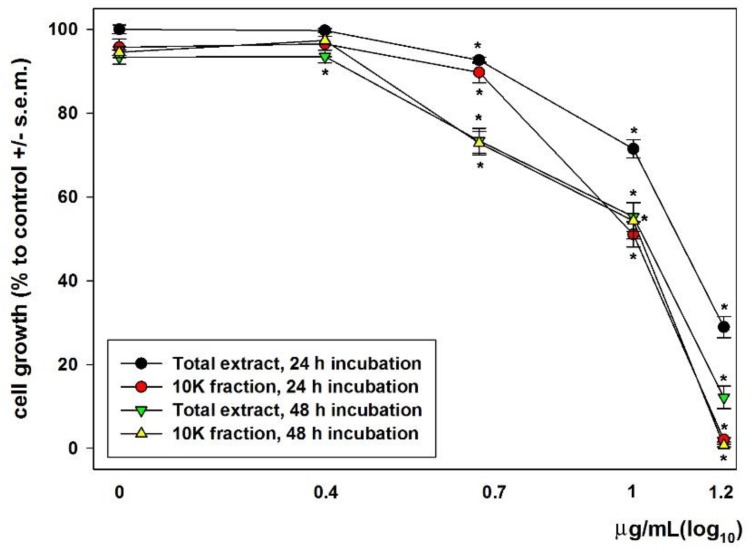
Dose response effect of total extracts and 10K fractions on the viability and growth of MDA-MB231 cells after either 24 or 48 h of exposure. Error bars correspond to the standard error of the mean (s.e.m.) of three independent measurements. * *p* < 0.05.

In a first set of experiments, we checked the effect of dose- and time-dependent incubation with total extracts and 10 K fractions on MDA-MB231 cell survival and growth via MTT assay. As shown in Figure 2, when cells were exposed for 24 h and 48 h to both samples, they underwent a concentration-dependent decrease of cell viability and proliferation. At the highest concentration tested, i.e., 15 μg/mL, the 10K fraction was more potent than the total extract in inhibiting cell viability and growth after both 24 and 48 h of incubation. The average IC_50_ was found to be 12.6 and 11.15 μg/mL at 24 h exposure and 10.1 and 10.45 μg/mL at 48 h exposure for the total extract and 10K fraction, respectively. Once the cytotoxicity of the two preparations on MDA-MB231 cells was confirmed, all the subsequent experiments were performed after cell exposures to total extracts and 10K fractions at the corresponding IC_50_ for 24 and 48 h. 

In order to get more insight into the biological basis of the cytotoxic activity exerted by the total extracts and 10K fractions on triple-negative MDA-MB231 cells, we checked their effects on cell cycle distribution, apoptosis induction via evaluation of phosphatidylserine externalization, mitochondrial transmembrane potential (MMP) state, ROS production, and autophagy modulation, the latter via quantitation of the acidic vesicular organelles (AVOs) and of specific intracellular biochemical markers, i.e., beclin-1, total LC3 and LC3-II.

**Figure 3:**
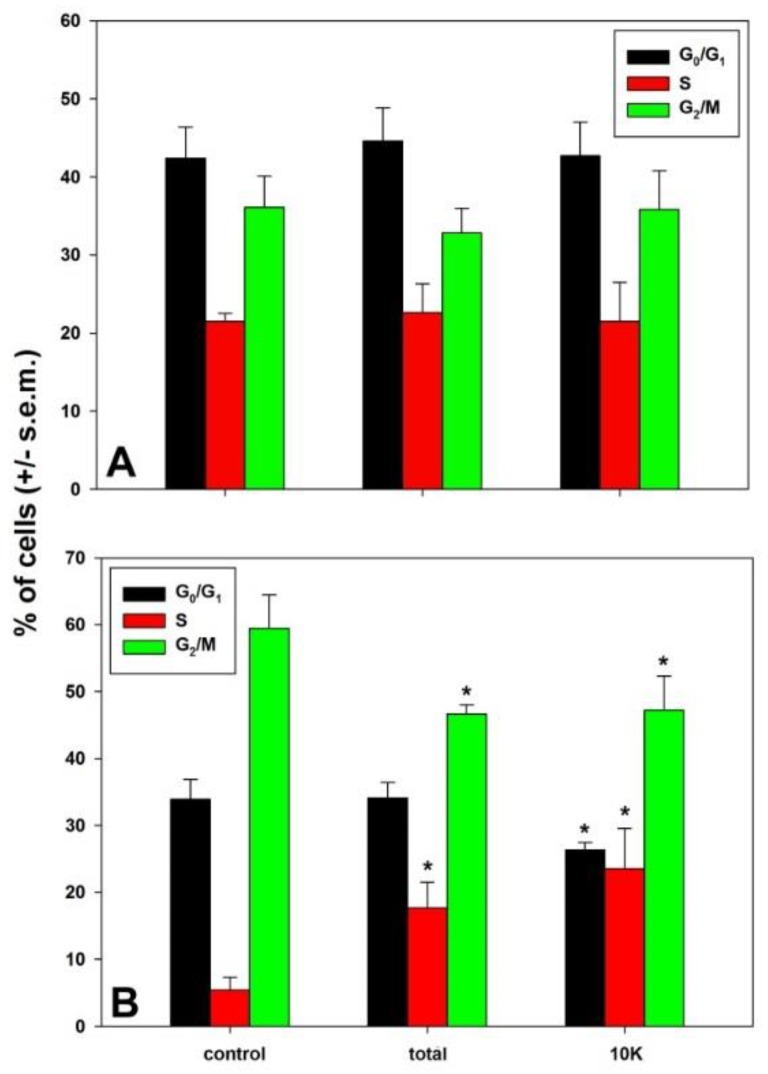
Cell cycle distribution of MDA-MB-231 cells exposed to total extracts and 10K fractions for 24 (**A**) and 48 h (**B**), compared to control conditions. Error bars correspond to the standard error of the mean (s.e.m.) of three independent measurements. * *p* < 0.05.

First, we tested which kind of perturbation was induced by treatments on MDA-MB231 cell cycle. Figure 3 shows that, when compared to the control, 48 h exposure to both preparations induced an accumulation of cells in S phase fraction, more conspicuous for 10K fraction (control vs. total extract vs. 10K fraction = 5.43% vs. 17.7% vs. 23.5%), and a decrease of G_2_/M fraction (control vs. total extract vs. 10K fraction = 59.48% vs. 46.6% vs. 47.2%). In addition, the 10K fraction determined a decrease also of the G_0_/G_1_ fraction (control vs. 10K fraction = 33.91% vs. 26.53%), which was not observed after exposure to the total fraction. No modification of cell distribution in the cycle phases was recorded at 24 h exposure to both preparations with respect to control.

**Figure 4:**
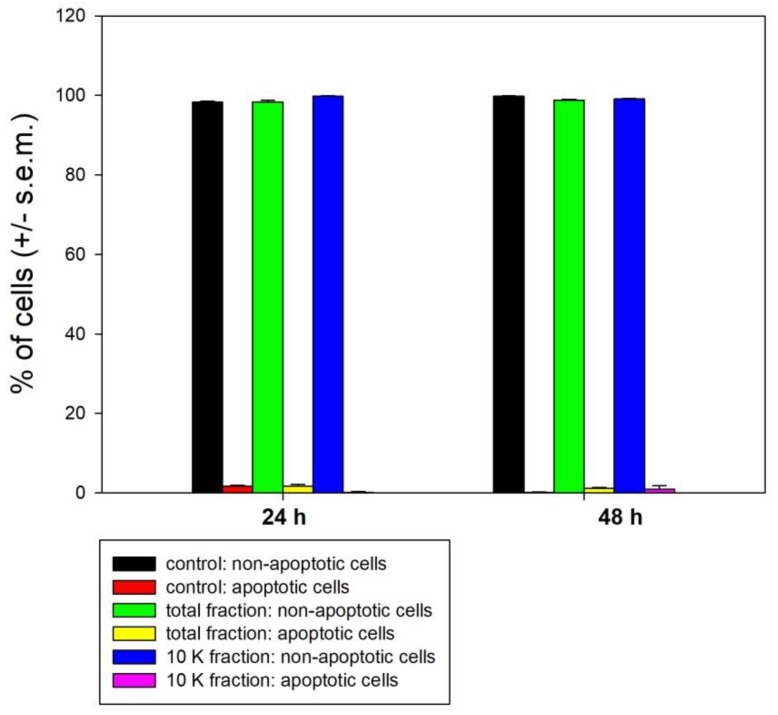
Histograms showing the percentage of apoptotic (annexin V+/propidium iodide−) vs. non-apoptotic MDA-MB-231 cells cultured in control conditions or exposed to either total extracts or 10K fractions for 24 and 48 h. Error bars correspond to the standard error of the mean (s.e.m.) of three independent measurements. * *p* < 0.05.

Samples of control and treated cells were then submitted to annexin V-FITC/propidium iodide staining in order to test the effect of the total extract and the 10K fraction on phosphatidylserine externalization, as a hallmark of induction of apoptosis, after 24 and 48 h of treatment. The data obtained showed no difference between control and exposed cells with the almost total absence of apoptotic annexin+/propidium iodide− cells at all times of investigation (Figure 4), demonstrating that programmed cell death was not involved in cytotoxicity. 

**Figure 5:**
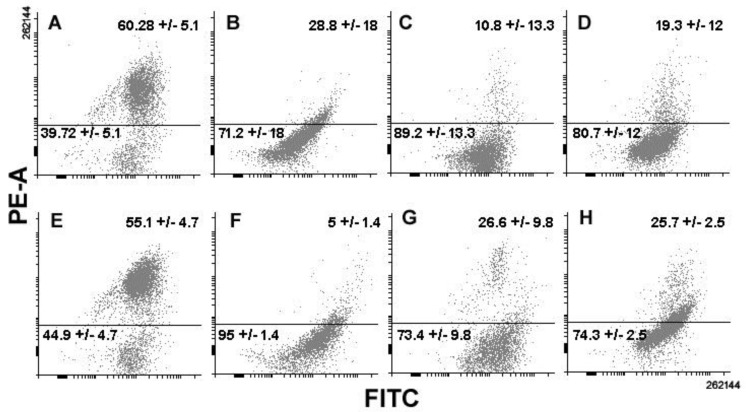
Representative flow cytometric analyses of the transmembrane mitochondrial potential (MMP) in control (**A**,**E**), total extract- (**C**,**G**), and 10K fraction-treated (**D**,**H**) MDA-MB231 cells for 24 (**A**–**D**) and 48 h (**E**–**H**). Dot plots (**B**) and (**F**) show the parallel positive controls of valinomycin-treated cells. The values indicated in the bottom quadrants in each frame quantitate the percentage of low red-emitting cells that underwent dissipation of MMP. The results are expressed as mean ± s.e.m. of triplicate assays.

The possible impairment of the mitochondrial function was evaluated by checking with the JC1 probe the MMP status following cell exposure to the preparations. As shown in Figure 5, flow cytometric analyses reveal a loss of the MMP in treated cells, the percentage of low red-emitting cells (bottom quadrant) being about 89% and 81% after 24 h exposure and about 73% and 74% after 48 h exposure to total extracts and 10K fractions, respectively, vs. approximately 40% and 45% in control cells in the two time intervals. 

**Figure 6:**
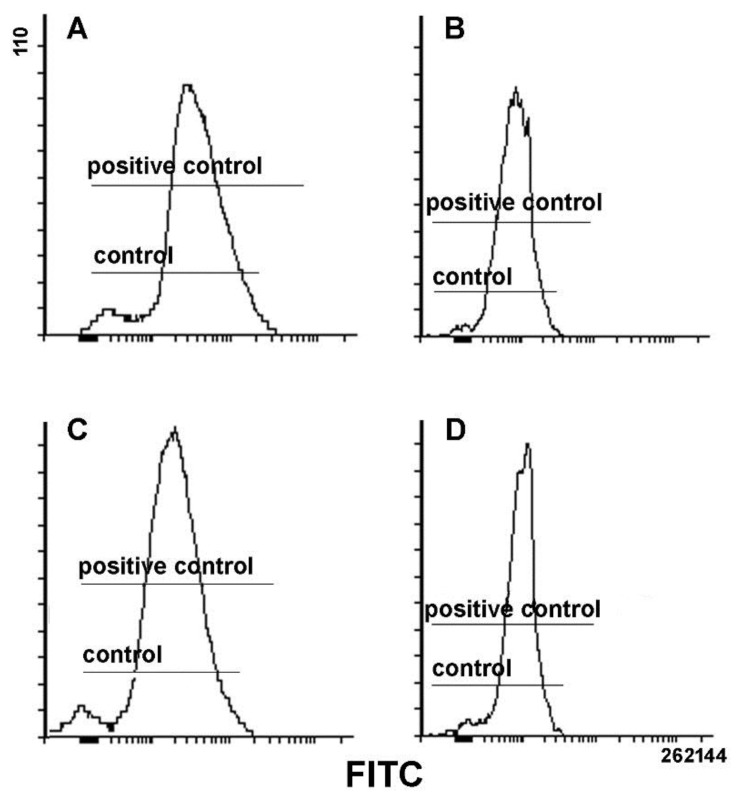
Representative plots showing reactive oxygen species (ROS) accumulation in gated alive MDA-MB231 cells exposed to total extracts (**A**,**C**) and 10K fractions (**B**,**D**) for 24 (**A**,**C**) and 48 h (**B**,**D**), compared to controls and H_2_O_2_-treated cells as positive controls. The *x*-axis reports the intensity of green fluorescence emitted by the dye 2’,7’-dichlorodihydrofluorescein, which is proportional to the amount of cytosolic ROS.

The imbalance of cell redox state was assayed via evaluation of ROS production. As shown in Figure 6, after gating to exclude the population of low-fluorescence-emitting dead cells from the analysis, the results obtained indicate that both 24 and 48 h exposure to total extracts and 10K fractions did not result in the overaccumulation of total intracellular ROS (superoxide anion, hydrogen peroxide, hydroxyl radical and singlet oxygen), being the fluorescence intensities comparable to those of untreated controls and smaller than those of positive controls.

**Figure 7:**
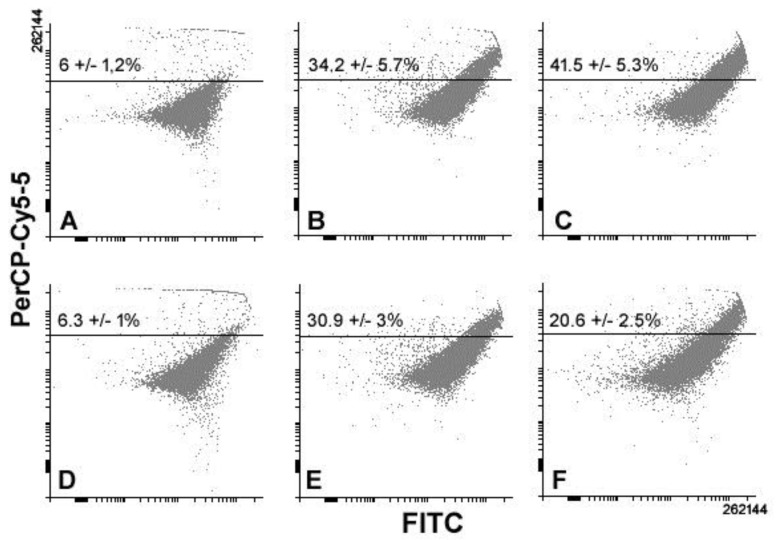
Representative flow cytometric analyses of control (**A**,**D**), total extract- (**B**,**E**), and 10K fraction-treated (**C**,**F**) MDA-MB231 cells stained with acridine orange for evaluation of acidic vesicular organelle (AVO) accumulation after 24 (**A**–**C**) and 48 h (**D**,**F**) of exposure. The percentage indicated in the top quadrants refers to the high red fluorescence-emitting events. The results are expressed as mean ± s.e.m. of triplicate assays.

Lastly, we checked whether the CF preparations could exert an effect on the modulation of the autophagic activity by TNBC cells. A first set of assays was performed to evaluate the possible modification in the amount of AVOs, a hallmark of autophagy, through staining with acridine orange, a cell-permeable fluorescent dye that can be captured within the acidic compartments after uptake and protonation. Interestingly, although as already reported, the autophagy rate of the cell line and consequently the amount of red-emitting MDA-MB231 cells are constitutively high [18,19,22]. Figure 7 shows that a further increase of fluorescent events can be observed after exposures to the preparations for 24 and 48 h, being more conspicuous in the presence of the 10K fraction upon 24 h of treatment. 

It is widely recognized that acridine orange is a reliable marker for monitoring autophagy only when used in conjunction with other molecular markers [23]. For this reason, to confirm the acridine orange data, we then checked the intracellular accumulation levels of beclin-1, whose increase is a hallmark of the onset of autophagy, and LC3-II, the autophagosome-linked lipidated form of LC3-I protein, whose amount is closely correlated with that of the organelles, thereby being a good indicator of their formation. For LC3-II quantification, unfixed cells were resuspended in saponin-containing buffers before the flow cytometric immunoassays in order to extract and discard the amount of immunoreactive soluble cytosolic LC3-I [21]. In addition, since conflicting evidence exists regarding the upregulation of LC3 transcription levels in autophagy-leading conditions of cellular stress [24,25,26], we also examined the effect of MDA-MB231 cell exposure to total extracts and 10K fractions on the intracellular accumulation of total LC3 via flow cytometric immunoassays performed on fixed cells.

**Figure 8:**
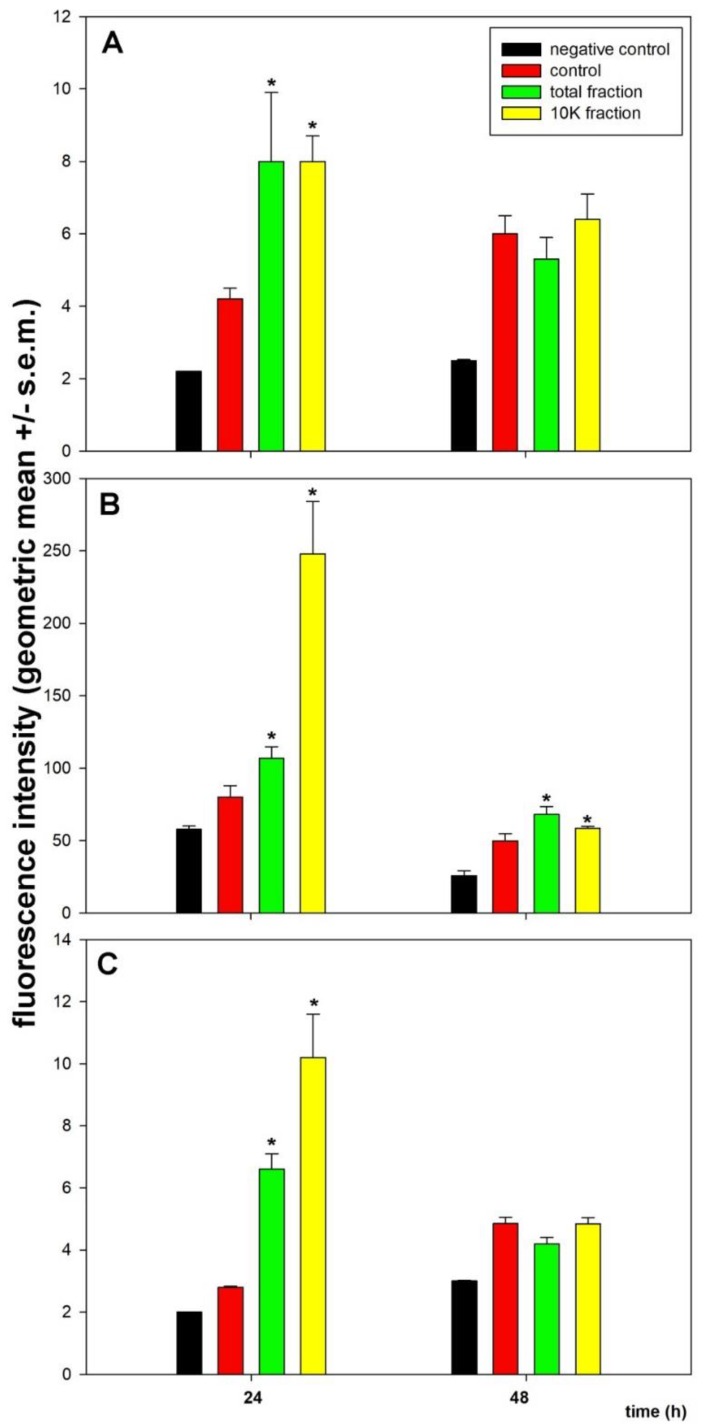
Histograms showing the intensity of the immunofluorescence of beclin-1 (**A**), LC3-II (**B**), and total LC3 (**C**) in control and treated MDA-MB-231 cells after 24 and 48 h of exposure. Negative controls lack the incubation with the primary antibody. Error bars correspond to the standard error of the mean (s.e.m.) of three independent measurements. * *p* < 0.05.

The histograms in Figure 8 confirm that MDA-MB231 cells have a high basal autophagy level and that an accumulation of autophagy markers may be observed after exposure to the CF preparations. In fact, control cells display a 1.4- to 2-fold higher LC3-II-related fluorescence intensity than the negative controls, thus indicating a constitutive activation of autophagosome formation in unexposed conditions. On the other hand, the amount of beclin-1 is further upregulated by about 1.9-fold in the presence of both total extract and 10K fraction after 24 h of treatment, being instead comparable to that of the control at 48 h of exposure. Also, the LC3-II amount further increases after 24 h of treatment, more prominently in the presence of the 10K fraction (10K fraction: About 3.1-fold; total fraction: 1.3-fold vs. control), whereas after 48 h, although remaining upregulated, it displays a more moderate increase of fluorescence in 10K fraction-exposed cells (10K fraction: About 1.4-fold; total fraction: 1.2-fold vs. control). Moreover, flow cytometric immunoassays for total LC3 show a greater intracellular accumulation of the whole amount of the protein at 24 h of exposure to both preparations, being more conspicuous in the presence of the 10K fraction (10K fraction: About 3.6-fold; total fraction: 2.35-fold vs. control), likely due to an early transcriptional activation. After 48 h of exposure to the CF preparations, total LC3 amount is comparable to that of control cells. Cumulatively, our results suggest the occurrence of an early autophagy induction upon cell exposure to CF extracts, followed by a flux decline approximately down to the levels of control samples between 24 and 48 h of treatment.

## 4. Discussion

Breast cancer, the most common diagnosed tumour among females, is a widespread neoplastic histotype accounting for about 12% of all cancers, whose rates are increasing worldwide [27]. Approximately 15% of breast cancers are of the TNBC subtype, which lacks effective targeted therapies, thereby being regarded as the most aggressive form of breast carcinomas to date [28]. In the attempt to identify novel substances endowed with anti-breast cancer action, here we have tested the cytotoxic effect of aqueous extracts of CFs from *H. tubulosa* on MDA-MB231 TNBC cells, also comparing the activity of the crude extracts to that of the filtered counterparts retaining peptides and other water-soluble components with MW < 10 kDa. Our overall results indicate that although the two preparations play the same biological role, the 10K fraction displays a lower IC_50_ at 24 h and appears more powerful in acting upon the mitochondrial function and the autophagy rate at 24 h, thus suggesting that the component(s) responsible for such early effects may be more concentrated in the filtered samples. 

To our knowledge, here we report the first data linking cell exposure to *H. tubulosa*-derived extracts to the modulation of the autophagic event. In particular, our results on the initial biological effects that occur 24 h after cell exposure to the CF extracts highlight autophagy upregulation and MMP dissipation, but with undetectable ROS overproduction, which remains unchanged thereafter. Autophagy, which is a cellular homeostatic function, implicates the autodigestion of cytoplasmic substrates for their removal or turnover after sequestration in the autophagic vacuoles, multi-membrane-bound structures, that then fuse with lysosomes generating AVOs or autolysosomes [29]. The role of autophagy in breast cancer is complex and controversial, since it may operate as a tumor-suppressor mechanism addressing neoplastic cells to death, but, on the other hand, it can act to protect cancer cells in conditions of oxygen and nutrient shortage and allow their rapid proliferative rate by providing energy and basic elements [30]. MDA-MB231 cells are endowed with a constitutively elevated autophagy rate. Inhibition of autophagy was proven to render them susceptible to the lethal effect of chemicals in various experimental conditions (e.g., [18,19,31,32]); on the other hand, lethal effects on this cell line induced by several autophagy inducers have been reported in the literature (e.g., [33,34]).

In this paper, the ability of the preparations under study to modulate the autophagic rate was checked via two different endpoints, i.e., the flow cytometric evaluations of the intracellular accumulations of AVOs and of the protein markers LC3 (total and LC3-II form) and beclin-1, the latter being a key component of the autophagic machinery belonging to the signal-initiating class III phosphatidylinositol-3 kinase complex [35]. Interestingly, the collective data presented here demonstrate that the CF extracts determine autophagy upregulation as indicated by the increase in AVOs, beclin-1, LC3-II, and total LC3, similarly to what was observed for MDA-MB231 in other experimental conditions (e.g., [36,37]). The decrease of the levels of beclin-1 and LC3-II approximately down to those of controls at 48 h of exposure may reflect their intra-autophagosomal degradation by lysosomal hydrolases after the formation of autophagosomes, whereas that of total LC3 may be due to a decrement in protein synthesis [25,38]. However, it is worth mentioning that acridine orange may also detect lysosomes and is thus not specific to autolysosomes, and assessment of beclin-1 and LC3-II levels is one of the most commonly used markers for autophagy, but as suggested by Klionsky et al. [23], should be coupled to other markers to give exhaustive indication of autophagic activity. Therefore, further studies are required to accurately determine whether autophagy is affected and autophagic activity is modified in CF extracts-treated MDA-MB231 cells.

It is known that treatment of MDA-MB231 cells with compounds that induce mitochondrial dysfunction and loss of MMP selectively results in the activation of autophagy, and that the originated autophagosomes are mainly addressed to engulf the damaged organelles via binding through an endogenous protein complex that contains the proteins LC3-II and Mitofusin 2 [39]. Therefore, our results suggest that the upregulation of autophagy observed after cell exposure to the CF preparations might be, at least in good part, of the “mitophagic” type. This hypothesis is substantiated by the absence of activation of cell death program, as observed after cell exposure to CF preparations. In fact, it is also known that autophagosomal degradation of the damaged mitochondria reduces the intracytoplasmic release of cytochrome C and inhibits the onset of intrinsic apoptosis [32]. It must also be taken into consideration, however, that MDA-MB-231 cells are provided with a mutant p53 and high levels of phospholipase D, both sustaining survival, thereby being intrinsically resistant to apoptosis [40,41]. Moreover, the autophagic clearance of the damaged mitochondria can also determine an interference with ROS production that might account for the inability to detect their increased accumulation in our experimental conditions, as found in other cases of MMP collapse in MDA-MB231 cells [18,19,31]. Interestingly, Gonzalez et al. [42] have reported the occurrence of a cytoprotective antioxidant response in MDA-MB231 cells induced by ROS-triggered autophagy upregulation, which implies the onset of a feedback loop between the free radical-protecting Keap1-Nrf2 pathway and the autophagy-related protein Atg7. 

Treatment with both CF preparations was shown to exert a selective effect on cell cycle distribution in a later timeframe, i.e., between 24 and 48 h of exposure, by producing a significant accumulation of MDA-MB231 cells at the S phase. It is conceivable that cells, increasingly damaged during the early times of exposure to the CF extracts, in this time lapse start to meet failures in DNA replication activity with a consequent slowing down of the specific cycle phase, if not an arrest, compared to controls [43]. It is worth mentioning that substances that promote autophagy are also known to alter the progression of the cell cycle preferentially in the S phase (e.g., [44,45]). Interestingly, the restraining property of the CF extracts from *H. tubulosa* reported here is shared with the aqueous extracts from another Mediterranean Sea cucumber, i.e., *Holothuria polii*, which were found to arrest MDA-MB231 cells in the S phase after three days of exposure [46].

Further elucidation of these and other autophagy-related molecular circuits through which the extracts of *H. tubulosa* CF accomplish their TNBC cell death activity will be carried out in future studies. Nonetheless, our work presents interesting aspects in that it expands the list of the preparations of natural origin that upregulate autophagy, while inhibiting tumor cell viability and provides more insight into the role played by autophagy in breast cancer cell death. It is indeed known that other echinoderms, including the Holothurian species, produce small molecules or amino acids that are able to induce autophagy in cancer cells [47,48,49].

A limitation of this study is the lack of identification of the CF component(s) responsible for the observed effects, which has/have not been isolated to date. On the other hand, the data reported here strongly suggest that such water-soluble constituent(s) is/are resistant to lyophilization, resuspension, and freeze-thawing cycles, and must supposedly have a MW < 10 kDa since the biological activity is retained and ameliorated after filtration of the crude extract. In light of the literature data, sea cucumber-derived anti-cancer molecules that exhibit this size fall into the categories of triterpenes, glycosaminoglycans, and peptides [8,50]. At present, we cannot exclude the existence of potential synergic activities, as reported by Guo et al. [51]. 

A great deal of literature data have widely discussed the molecular nature of potential anti-cancer compounds obtained from sea cucumbers [52]. Notably, the majority of them consist of molecules recovered after fractionation of extracts in organic solvents, and hence our work adds one more reference to the limited number of publications that instead focus on the cytotoxic activity of aqueous extracts and their components (e.g., [46,53,54]).

## 5. Conclusions

In summary, we have shown that extracts from *H. tubulosa* CF are cytotoxic towards TNBC cells in vitro and therefore merit further investigation aimed at the characterization of the “active ingredient(s)” present in the samples under study to develop novel promising prevention and/or treatment agents efficacious against highly metastatic breast carcinomas.
